# Preparation of a new type 2 diabetic miniature pig model via the CRISPR/Cas9 system

**DOI:** 10.1038/s41419-019-2056-5

**Published:** 2019-10-28

**Authors:** Xiaodong Zou, Hongsheng Ouyang, Tingting Yu, Xue Chen, Daxin Pang, Xiaochun Tang, Chengzhen Chen

**Affiliations:** Jilin Provincial Key Laboratory of Animal Embryo Engineering, College of Animal Sciences, Jilin University, Changchun, Jilin Province People’s Republic of China

**Keywords:** Biotechnology, Genetic engineering

## Abstract

Diabetes has become one of the major noninfectious diseases that seriously endanger public health. The formation of islet amyloid polypeptide (IAPP) affects the normal physiological functions of the body, such as glucose metabolism and lipid metabolism. The mature human IAPP protein (hIAPP) has a strong tendency to misfold and is considered to be one of the major causes of amyloid changes in islets. Deposition of hIAPP is considered to be one of the leading causes of type 2 diabetes mellitus (T2DM). Miniature pigs are experimental animal models that are well suited for research on gene function and human diabetes. In our study, we obtained *IAPP* gene-humanized miniature pigs via the CRISPR/Cas9 system and somatic cell nuclear transfer (SCNT) technology. The hIAPP pigs can be used to further study the pathogenesis and related complications of T2DM and to lay a solid foundation for the prevention and treatment of T2DM.

## Introduction

Diabetes mellitus (DM) is one of the most widespread chronic diseases worldwide. Diabetes causes frequent urination, thirst, hunger, and weight loss, which are the most common features of diabetes^1^. Polyuria, polydipsia, polyphagia, and weight loss are the most common clinical symptoms in diabetic patients^2^. In 2010, 285 million adults suffered from diabetes, and the world prevalence of diabetes was 6.4%, and the prevalence is predicted to increase to 7.7% (439 million adults) by 2030^3^. According to reports, the mortality rate of diabetes, including all age groups, is approximately 3.96 million people per year^4^. According to the WHO-approved classification method, diabetes can be broadly divided into four categories: type 1 DM, type 2 diabetes mellitus T2DM), gestational DM, and other special types of diabetes^5^.

In diabetics, 90% of patients with diabetes are diagnosed with T2DM^3^. T2DM, described as a silent disease, is characterized by insufficient insulin secretion and insulin resistance^6^. The main symptoms of T2DM include dyslipidemia, hyperglycemia, hypertension, and atherosclerosis^7^. As of 2015, approximately 392 million people were diagnosed with the disease, compared with approximately 30 million people in 1985^8^. According to reports, the aging of the global population, reduced exercise, and increased obesity are the main reasons for the increase in diabetes^9^. Previous studies have shown that T2DM is an adult disease, but it has been increasingly diagnosed in obese children in recent years^10^. It is believed that by 2030, T2DM will be the seventh leading cause of death in the world^11^.

Islet amyloid polypeptide (IAPP, amylin) was discovered, extracted and named from islet tumor cells and is a polypeptide hormone containing 37 amino acid residues; IAPP is secreted by islet beta cells^12^. It is mainly stored in the halo of the secretory granule, secreted in a pulsed manner under the action of glucose and other secretagogues^13^. Studies have shown that IAPP has a physiological role in glucose metabolism^14^ and lipid metabolism^15^. In addition, it also has a toxic effect on β cells^16^. In brief, IAPP forms oligomers that cause beta cell apoptosis, thereby promoting islet amyloidosis, which ultimately leads to the progressive failure of insulin secretion^17^. In the process of glucose metabolism, the role of IAPP is mainly reflected in the inhibition of glycogen synthesis, glucose transport, glucose uptake in muscle tissue and the utilization of glucose by hepatocytes^18^. Moreover, it also increases the output of hepatic glycogen^19^. Its effects on lipid metabolism are still unclear^20^. The IAPP protein is soluble, and its single subunit state is unfolded. It can synergize with blood glucose-regulating hormones, such as insulin, to more precisely regulate human blood sugar^21^. However, the mature human IAPP protein (hIAPP) has a strong tendency to misfold and can form amyloid aggregates. hIAPP is one of the most highly aggregated polypeptides of 20 amyloid aggregated peptides that have been discovered thus far^22^. Some studies have reported that islet amyloid deposition was found in islets of T2DM patients. hIAPP amyloidosis is considered to be a potentially important cause of T2DM^23^.

Pigs are model animals that are closest to humans except for nonhuman primates^24^. Pigs and humans are very similar in terms of anatomy, physiology and biochemical metabolism^25^. Pigs have the advantages of early sexual maturity, short timespan between generations, high number of litters, and highly precise genetic modification^26^. Pigs can be used to study surgical and standard diagnostic techniques in human medicine; in turn, new diagnostic methods that have proven effective in pigs can also be used directly in humans^27^. Similar to humans, pigs are omnivores, and have similar dietary needs and nutritional balance requirements^28^. The time required for intestinal food digestion and transformation is also closest to that in humans^29^.

At present, there is no recognized ideal animal model that is completely consistent with the characteristics of human T2DM^30^. Worldwide, studies have shown that miniature pigs are ideal animal models for studying human obesity and metabolic-related diseases^31^. New animal models are needed, especially large animal models, to bridge the gap. Therefore, the establishment of the hIAPP miniature pig model is of great significance for the study of the pathogenesis and complications of T2DM.

## Results

### Amino acid sequence homology analysis of IAPP mature peptides of various species

**Fig. 1:**
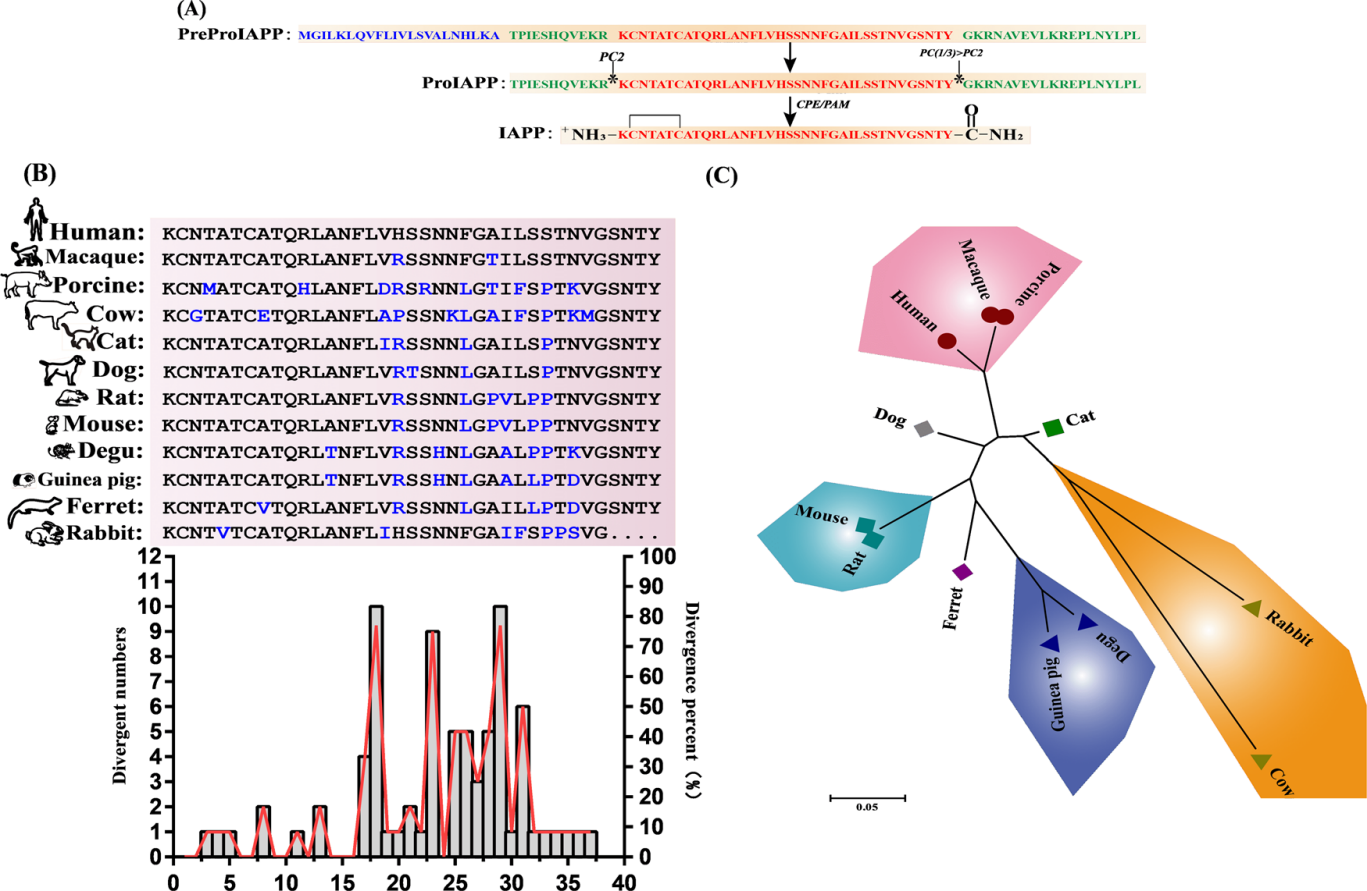
Process of human IAPP precursor processing to form mature IAPP and homology comparison of IAPP mature proteins in different species. **a** Human IAPP was processed to form mature IAPP. The signal peptide consisting of 22 amino acid residues is represented by blue, the flanking regions of the original N-terminal and C-terminal regions of IAPP are indicated by green, and the mature peptide sequences are indicated by red. **b** Comparison and analysis of amino acid sequence homology of mature IAPP proteins between different species. **c** Unrooted tree of IAPP mature protein between different species

The human IAPP gene is located on the short arm of chromosome 12 and belongs to the calcitonin family. The 89-residue IAPP precursor (PreproIAPP) was first synthesized in islet β cells. Then, PreproIAPP is hydrolyzed by signal peptidase in the endoplasmic reticulum to form a 67-residue ProIAPP, which is digested by the prohormone convertase enzyme 1/3 and PC2 in the Golgi. After entering the secretory vesicle, it is further decomposed by carboxypeptidase E (CPE) and amidated monooxygenase (PAM) to form a mature IAPP consisting of 37 amino acids^32^ (Fig. 1a). The mature IAPP peptides between different species have high homology. The amino acid sequence of the IAPP mature peptide between humans and other species differs mainly at positions 18, 23, 29, and 31 (Fig. 1b). Subsequently, the amino acid sequence alignment results revealed that the mature porcine IAPP peptide (95%) was more homologous to the human peptide (Fig. 1c).

### Alignment of pig-to-human mature IAPP peptide sequence and construction of the IAPP-humanized CRISPR/Cas9 system

**Fig. 2:**
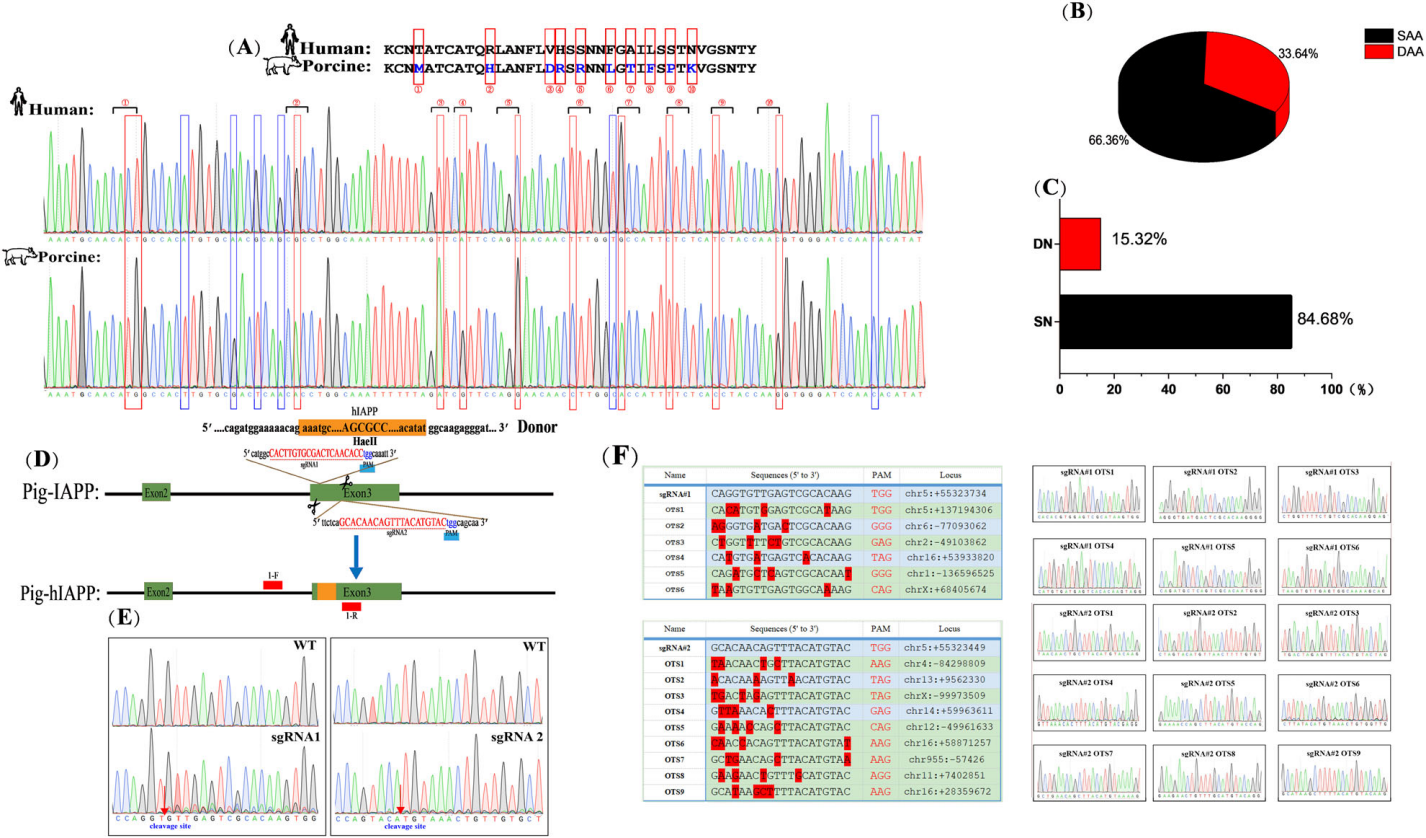
Sequence analysis of mature pig-to-human IAPP protein and construction of the humanized IAPP gene CRISPR/Cas9 system. **a** Sequence homology comparison between pig and human IAPP mature protein. The red box marks the different nucleotide sequences of the porcine and human IAPP mature proteins. The blue box marks the nucleotide sequence that does not cause amino acid changes. **b** A pie chart of amino acid differences between the pig and human mature IAPP proteins. The red part indicates the percentage of differential amino acids (33.64%); the black part indicates the percentage of the same amino acids. **c** A nucleotide difference pie chart corresponding to the mature IAPP protein of pigs and humans. The red part indicates the percentage of differential nucleotides (15.32%); the black part indicates the percentage of the same nucleotides (84.68%). **d** Schematic representation of sgRNAs to porcine IAPP exon 3. sgRNA targeting sequences are shown, and PAMs are highlighted in blue. **e** Genomic sequences of CRISPR target regions in wild-type PFFs and transfected PFFs, as indicated. Cleavage sites are labeled with red arrows. **f** Off-target detection. The sequencing peaks showed that both sgRNAs were not cleaved at other sites

Mature human and porcine IAPP peptide nucleotide sequences were amplified and sequenced, and the sequences of differences between hIAPP and pIAPP were confirmed by alignment (Fig. 2a). The results of the analysis indicated that the divergence in the nucleotide sequence of the IAPP mature peptide (33.64%) was higher than that of the amino acid sequence (15.32%) (Fig. 2b, c). We designed three sgRNAs targeting exon 3 of pIAPP (Fig. 2d). Subsequently, the cutting efficiency was detected, and two sgRNAs had high efficiency (Fig. 2e). Moreover, off-target detection was performed, and sequencing results showed that no cutting occurred at other sites (Fig. 2f, Supplementary Table 1).

### The construction process of the mature humanized IAPP peptide in miniature pigs

**Fig. 3:**
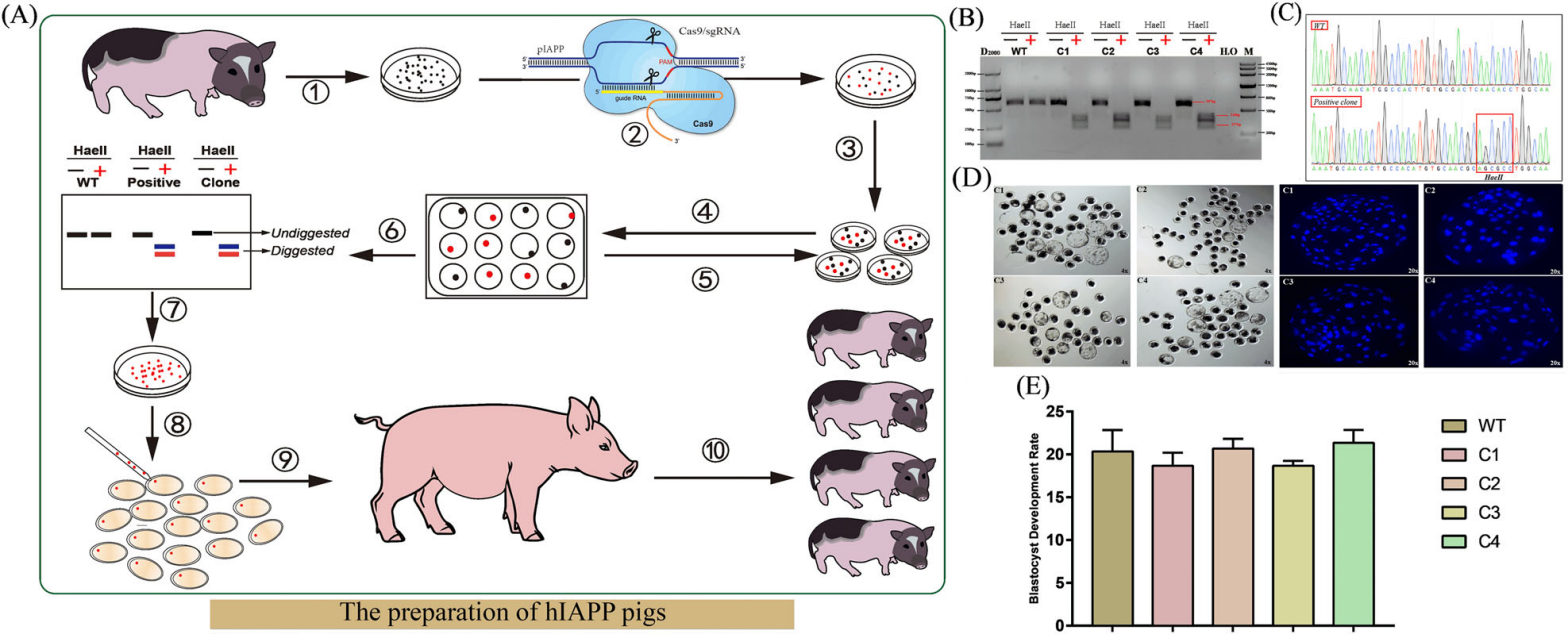
The preparation of hIAPP miniature pigs. **a** Experimental flow of IAPP-humanized pig construction. ① Isolation and culture of miniature PFFs; ② Transfection of miniature PFFs; ③ Inoculate cells into 10 cm culture dishes using the limiting dilution; ④ Monoclonal cell picking; ⑤ Repeat step ④; ⑥ Positive colonies were screened by restriction enzyme digestion assay; ⑦ Expansion of culture-positive clones; ⑧ Somatic cell nuclear transfer; ⑨ Embryo transfer; ⑩ Identification of hIAPP cloned pigs. **b**, **c** Positive cloned cells were identified by restriction enzyme digestion assay and verified by Sanger sequencing. **d** Statistics of blastocyst development were performed on 4 positive clones. 8.5 days after nuclear transfer, the cells that could develop normally into blastocysts, and the results of nuclear staining also prove this conclusion. **e** Statistical analysis of the blastocyst development rate of positive cloned cells. No significant differences were found compared to the control group

Figure 3a is a flow diagram of the construction of the mature humanized IAPP peptide miniature pigs (hIAPP pigs). First, four hIAPP-positive cell clones were identified by cell monoclonal techniques, Sanger sequencing, and HaeII digestion (Fig. 3b, c). Subsequently, the ability of the four positive cloned cells to develop blastocysts was tested. After 8.5 days of nuclear transfer, the number of cells that developed to the blastocyst stage was counted and stained (Table 1, Fig. 3d, e). Statistical analysis showed that there was no significant difference in the blastocyst development rate compared with that of the control group, and all of them could develop normally.

**Table 1:** Statistical results of the blastocyst development rate

Repeat times of SCNT	Type of donor Cell	Nuclear donor cell number	Blastocyst number	Blastocyst development rate (%)
1	C1	100	17	17
	C2	100	20	20
	C3	100	19	19
	C4	100	21	21
	WT	100	23	23
2	C1	100	19	19
	C2	100	22	22
	C3	100	18	18
	C4	100	23	23
	WT	100	18	18
3	C1	100	20	20
	C2	100	20	20
	C3	100	19	19
	C4	100	20	20
	WT	100	20	20

### Growth and development monitoring of hIAPP pigs

**Fig. 4:**
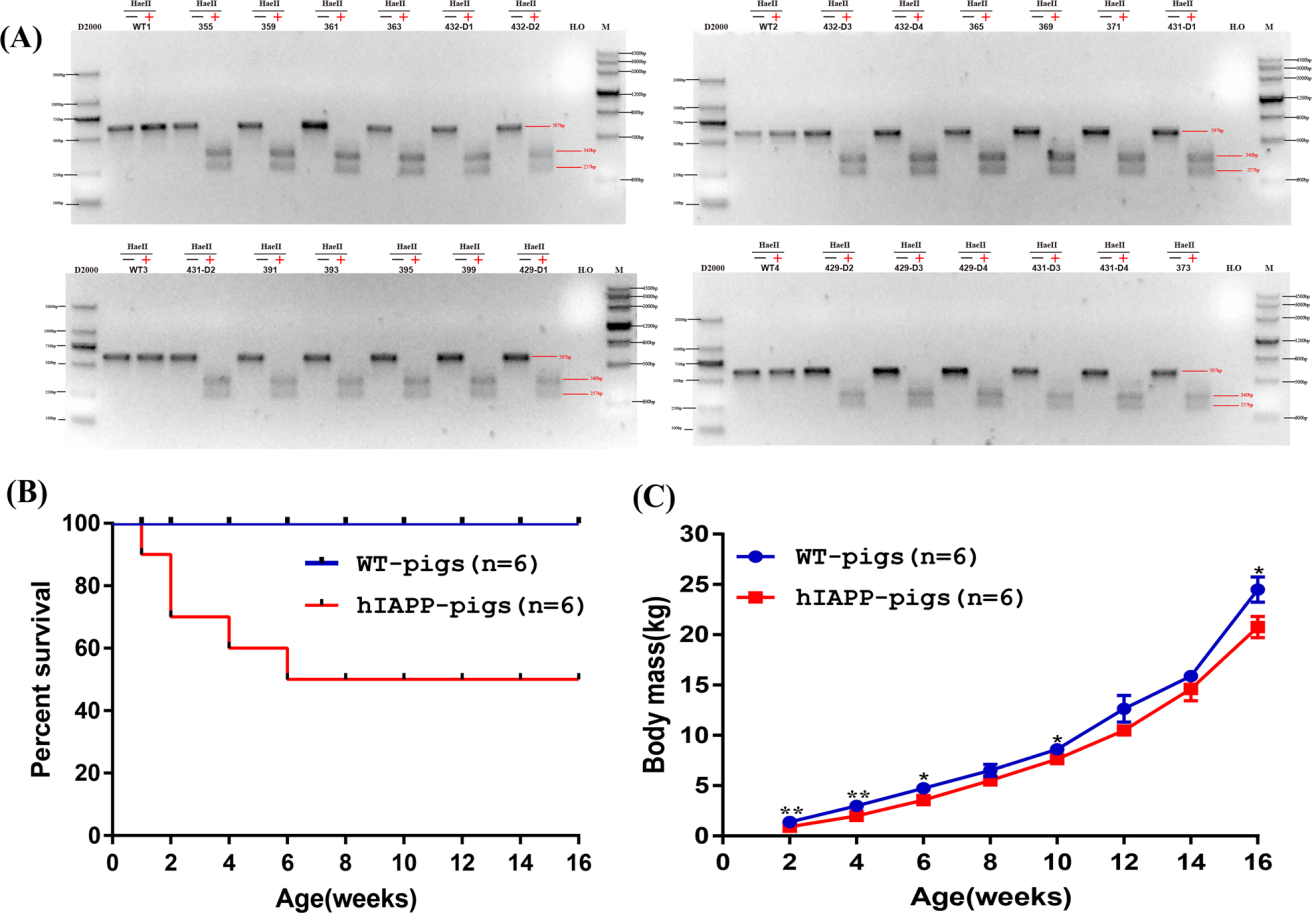
Generation and identification of hIAPP pigs. **a** hIAPP-positive pigs were identified by a restriction enzyme digestion assay and verified by Sanger sequencing. **b** Kaplan–Meier survival curves for the hIAPP pigs (*n* = 6) and WT pigs (*n* = 6). **c** Body mass comparison of hIAPP pigs (*n* = 6) and WT pigs (*n* = 6) from birth to 16 weeks of age. *P < 0.05, **P < 0.01, ***P < 0.001

Large white pigs were selected as surrogate sows, the number of embryos transplanted per surrogate sow was 300, and a total of 5 were transplanted. Four of the sows were pregnant, and after approximately 120 days of gestation, 28 piglets were eventually obtained (Table 2). After Sanger sequencing and HaeII digestion, 24 hIAPP-positive pigs were finally identified (Fig. 4a). Piglets with similar birth weights were selected for late weight monitoring. The hIAPP pigs and the control littermates were weighed weekly, and the data showed that the weight gain of the hIAPP pigs was slower than that of the control pigs (Fig. 4b). Approximately 33.3% of the hIAPP pigs died within the first 2 weeks after birth, and half (50%) died by 6 weeks of age (Fig. 4c).

**Table 2:** SCNT results for the generation of gene-targeted pigs

Surrogate ID	Positive clone cell ID	No. embryos transferred	Gestation length (days)	No. pigs born	No. pigs humanized
429	C1 + C2 + C3	300	/	/	/
430	C1 + C2 + C4	300	119	7	5
431	C2 + C3 + C4	300	120	6	6
432	C1 + C2 + C3	300	120	8	7
433	C1 + C2 + C4	300	121	7	6

### Phenotypic analysis of hIAPP pigs

**Fig. 5:**
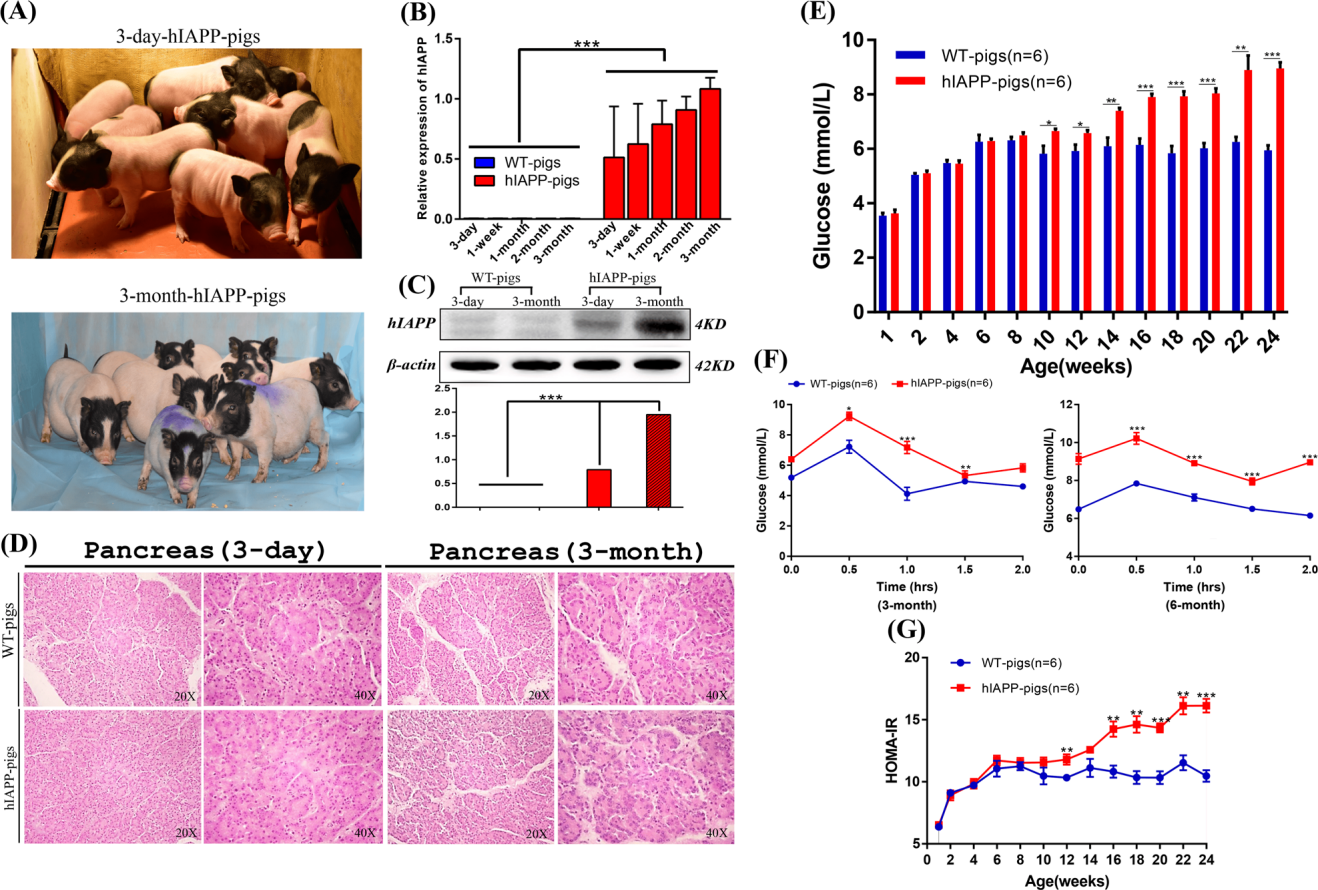
Detection of the phenotype of hIAPP pigs. **a** Photograph of hIAPP piglets. **b** The expression of hIAPP mRNA was detected in model pigs, and no expression of hIAPP mRNA was detected in the littermate control group, and the expression level of older model pigs was higher than that of younger model pigs. **c** Western blotting. The results showed that the human IAPP protein was detected in the IAPP-humanized cloned pigs, but the expression of the protein was not found in the control pigs. At the same time, the expression of hIAPP protein in the 3-month-old model pigs was higher than that of the 3-day-old model pigs. **d** H&E staining. Pathological analysis of pancreatic tissue sections revealed no lesions. Scale bars: 50 µm. **e** Fasting blood glucose levels of the hIAPP and WT pigs from 3 days to 24 weeks. *P* < 0.05 was considered statistically significant, *P < 0.05, **P < 0.01, ***P < 0.005. **f** Glucose tolerance levels in hIAPP and WT pigs at 3 and 6 months of age. *P* < 0.05 was considered to be statistically significant, *P < 0.05, **P < 0.01, ***P < 0.001. **g** The HOMA-IR comparison of hIAPP pigs and control groups. *P* < 0.05 was considered to be statistically significant, *P < 0.05, **P < 0.01, ***P < 0.001

The model pigs have good growth and development (Fig. 5a). The expression of the hIAPP protein at the transcriptional and translational levels was tested in the pancreas of hIAPP pigs. Real-time quantitative polymerase chain reaction (PCR) results showed that the expression of hIAPP mRNA was detected in model pigs, and no expression of hIAPP mRNA was detected in the littermate control group. Moreover, the expression levels of hIAPP mRNA were different in model pigs of different ages; the expression level in older model pigs was higher than that in younger pigs (Fig. 5b). This conclusion was also verified by Western blotting. The expression of the hIAPP protein in 3-month-old model pigs was higher than that in 3-day-old model pigs (Fig. 5c). Subsequently, we performed a pathological analysis of the hIAPP model pigs. Hematoxylin and eosin (H&E) staining showed that compared to the control group, the model pig group had no lesions in the pancreas (Fig. 5d). Blood samples from hIAPP pigs and wild-type pigs were collected every two weeks to determine the relevant physicochemical indexes of the blood. Statistical significance was observed in the level of fasting blood glucose when pigs reached 10 weeks of age, and hIAPP pigs had increased fasting blood glucose with an increasing trend and reached 8.95 ± 0.55 mmol/L at 6 months, showing the symptom of hyperglycemia (Fig. 5e). However, no significant difference was discovered in fasting insulin (Supplementary Fig. S1). Moreover, the results of the glucose tolerance test at 3 months and 6 months are shown in Fig. 5f. Compared with that of the control pigs, the glucose utilization rate of the hIAPP pigs decreased significantly (*P* < 0.05, *n* = 6). Moreover, the results of the homeostatic model assessment for insulin resistance (HOMA-IR) showed that from the age of 3 months, the IR index of hIAPP pigs was significantly higher than that of the wild-type pigs, indicating that the hIAPP pigs had reduced insulin sensitivity and increased insulin resistance (*P* < 0 05, *n* = 6) (Fig. 5g). Taken together, the 3-month-old hIAPP pigs showed hyperglycemia, decreased glucose utilization, and increased insulin tolerance, indicating successful T2DM modeling in hIAPP pigs. Although the hIAPP model pigs are now 6 months old, since diabetes is a chronic disease, most of which occurs during old age, it is necessary to spend more time monitoring the development and changes in their phenotype and the emergence of complications.

Overall, we successfully constructed and identified the hIAPP model pigs of T2DM.

## Discussion

Studies have reported that IAPP can act on the brain and exert physiological functions, such as inhibiting gastric emptying and suppressing the appetite^33^, and IAPP can promote insulin and other blood glucose-regulating hormones to synergize to more precisely regulate the blood sugar of the human body^34^. It has been reported that the oligomer of hIAPP is mainly composed of α-helical structures and can penetrate the cell membrane through the membrane and anchor to form a hollow tubular structure on the membrane, which leads to the formation of voids and nonselective ion channels in the cell membrane^35^. It leads to the leakage of electrolytes, such as Na+ and Ca2+, inside and outside the membrane, and the osmotic pressure is unbalanced^36^. At the same time, the structure can also cause the formation of vesicles, tubules or membrane defects in the cell membrane, destroying the coupling between cells, and eventually leading to cell apoptosis^37^. In addition, the mature IAPP fibers in the cells can also induce oxidative stress, which in turn can promote the production of amyloid deposits, forming a vicious circle, and can cause apoptosis, eventually causing T2DM^38^.

Studies have shown that nonhuman primates and cats can form islet amyloid deposits^39^; no islet amyloid deposits are found in cattle^40^, rodents^41^, or dogs^42^, and although amyloid deposits are found in degu, it comes from insulin formation rather than IAPP aggregation^43^. IAPP in ferrets and pigs can form islet amyloid precipitates, but the IAPP amyloidogenic ability is much lower in ferrets and pigs than in humans^44^; rabbits and hares have only a partial IAPP sequence^44^. Although many mouse models of diabetes research have been established using forward genetic and reverse genetic methods, a number of murine animal models have been used to study the pathogenesis of human diabetes and its complications^45^. However, the use of model mice does not address all phenotypes (such as insulin resistance, obesity, hyperinsulinemia, and hypercholesterolemia) that tend to cause a decrease in glucose tolerance and T2DM. Therefore, the use of rodent models to study the occurrence of complications in T2DM is not sufficient. New animal models need to be explored, especially large animal models, to bridge the gap between mouse models and human patients.

Pigs are the model animals that are the closest to humans except for nonhuman primates. In many ways, pigs are attractive model species that mimic human physiology and pathology^46^. Global studies have shown that a miniature pig is an ideal animal model for studying human obesity and metabolic-related diseases^47^. In terms of anatomy, physiology, and biochemical metabolism, pigs are very close to humans, and similar to humans, diabetic pigs ultimately develop atherosclerosis, which does not occur in rodents and other animals^48^. The anatomical analysis revealed that there was a difference in the output of the pancreatic duct system between pigs and humans, but the pancreatic exocrine and endocrine glands of pigs were similar to humans in terms of size, shape, body position and blood supply^49^. In both human and porcine species, endocrine cells are mainly found in Langerhans island, or as a single cell or small cell, as clusters distributed throughout the exocrine pancreas^50^. It should be emphasized that these two species have more uncertain islet structures than rodents, and humans and pigs are very similar in terms of the size and composition of islets and the distribution characteristics of different endocrine cells^51^. Pig and human insulin differ in only the 30th amino acid of the β-strand, and porcine insulin has been used to treat human diabetes for decades^52^. In addition, the amino acid sequences of incretin, glucose-dependent insulinotropic polypeptide and glucagon-like peptide-1 are highly conserved in humans and pigs. These hormones promote insulin secretion after nutrient digestion^53^.

So far, there is no recognized ideal animal model that is fully consistent with the characteristics of human T2DM. There is an urgent need to use modern biotechnology to establish a genetically modified miniature pig diabetes model to study the pathogenesis and preventive measures of diabetes and its complications. In our study, we obtained a model of the humanized IAPP gene from the CRISPR/Cas9 system, providing a new model for studying human T2DM and its complications, which can be used to elucidate the genetic phenotype of pathogenesis and the characteristics.

## Materials and methods

### Ethics statement

All animal studies were approved by the Animal Welfare and Research Ethics Committee at Jilin University, and all procedures were conducted strictly in accordance with the Guide for the Care and Use of Laboratory Animals. All surgeries were performed under anesthesia, and every effort was made to miniaturize animal suffering.

### Construction of CRISPR/Cas9 gene-editing system

The vector backbone, including U6-sgRNA and Cas9 expression elements, was purchased from Addgene (Plasmid #42230). First, complementary sgRNA oligonucleotides were synthesized, annealed and ligated to the BbsI sites of the backbone vector to construct the intact plasmid confirmed by Sanger sequencing analysis.

### Isolation and culture of porcine fetal fibroblasts (PFFs)

Thirty-three-day-old fetuses were separated from Bama miniature sows, and primary PFFs were isolated from these fetuses. After removing the head, tail, limb bones, and viscera, these fetuses were cut into small pieces. Then, small pieces were digested with a sterile collagenase solution and cultured in DMEM (GIBCO) supplemented with 15% fetal bovine serum (FBS) at 39 °C and 5% CO_2_ in a humidified incubator. Isolated PFFs were digested from the culture dishes with 0.1% (w/v) trypsin in Dulbecco PBS (GIBCO) and centrifuged at 1000 rpm for 5 min. Then, after removing the supernatant, PFFs were re-suspended and cultured in new culture dishes with 15% FBS in a humidified incubator. Cells at passage 1 were frozen in FBS containing 10% dimethyl sulfoxide (DMSO).

### Construction of *hIAPP* donor vector plasmid

The donor vector contained a 500 bp left homologous arm and a 500 bp right homologous arm. The HAs were amplified by genomic PCR of Bama miniature pigs and cloned into the PLB vector (Beijing, China). The hIAPP gene was synthesized by GENEWIZ (Suzhou, China) and inserted between the left and right arms.

### Electrotransfection and detection of CRISPR/Cas9 system editing efficiency

Electrotransfection was carried out according to the previous research^54^. First, 3×106 PFFs were electroporated with 200 μL of Opti-MEM (GIBCO) and 30 μg plasmids using 2 mm gap cuvettes of BTX ECM 2001. The parameters for electrotransfection were as follows: 3 pulses of 340 V for 1 ms repeated once. After 36 h of electrotransfection, the cells were digested with 0.25% trypsin, and the genome was extracted as a template for the detection of the cutting efficiency of the CRISPR/Cas9 gene-editing systems by PCR.

### Positive single-cell-colony selection

After determining the editing efficiency of the CRISPR/Cas9 system, high efficiency sgRNAs were selected for electrotransfection. During electrotransfection, 15 μg hIAPP donor plasmids and 15 μg CRISPR-sgRNA plasmids with 200 μL Opti-MEM were added. After 36 h of electrotransfection, the cells were plated into 20 dishes of 10 cm at a density of 5 × 103 cells per dish. After 8–9 days of culture, single-cell colonies were picked and cultured in 24-well plates. Twenty percent of each colony was lysed using 10 μL of lysis buffer (0.45% NP40 plus 0.6% proteinase K) for 60 min at 56 °C and then 10 min at 95 °C.

The lysate was used to detect cells from positive clones by PCR. The forward primer was 5′-CAGCTAAACAGAGTAAAGAG-3′, and the reverse primer was 5′-GATTTCCCTAGAGTCCACTT-3′. The PCR conditions were 94 °C for 5 min; 94 °C for 30 s, 55 °C for 30 s, and 72 °C for 40 s for 35 cycles; 72 °C for 5 min; and a hold at 16 °C. The PCR products were ligated into the PLB vector (Tiangen, Beijing, China) for sequencing. Cells from positive colonies were expanded and cryopreserved.

### Somatic cell nuclear transfer (SCNT) and embryo transfer (ET)

SCNT and ET were performed according to previous research^55^. Four positive colonies were screened and selected as donor cells for SCNT. The positive cell clones were injected into the perivitelline cytoplasms of enucleated oocytes. The reconstructed embryos were activated and cultured to develop into blastocysts. Blastocysts were stained with Hoechst 33342 for detection of cytotoxicity. High-quality blastocysts were transferred into synchronized recipient pigs.

### Genotyping of hIAPP piglets

To confirm the humanized IAPP gene, genomic DNA was extracted from the ears of piglets and used as the template for PCR using the 1F/1R primer pairs, as described above. Partial PCR products were subjected to electrophoresis and sequencing. Moreover, the rest of the PCR products were purified using a Normal DNA Purification Kit (DP204, Tiangen, China), and 2 μg of purified products were digested with Haeα for 2 h at 37 °C and identified by electrophoresis.

### Off-target assay

Highly similar sequences in the porcine genome were detected by BLAST, and potential off-target sites (OTS) were selected for each gRNA. All OTS were PCR amplified using the genomic DNA of the IAPP-humanized piglets as templates. Sanger sequencing was performed to examine off-target mutagenesis.

### Body weight and survival curve

The body weights of age- and sex-matched WT and hIAPP pigs were measured biweekly. A minimum of three individual animals of each genotype was used in all experiments.

### Quantitative real-time PCR

For the detection of the relative mRNA levels of the hIAPP gene, total RNA was isolated from pancreas samples. The reaction reagents were added following the manufacturer’s recommendations. The reaction conditions were 95 °C for 15 min; 95 °C for 10 s; 60 °C for 20 s, and 72 °C for 30 s for 40 cycles and 95–55 °C for 30 s (melting curve). The fluorescence intensity and amplification plots were analyzed by BIO-RAD iCycler Thermal Cycler w/ iQ5 Optical Module for RT-PCR (Bio-Rad, ABI 7500, iQ5). GAPDH was utilized as a reference gene. The primers used in RT-PCR are shown in the following table.

**Table:** 

RT-hIAPP-F (5′–3′)	CTGGAGCGTGGAGGAGAAC
RT-hIAPP-R (5′–3′)	TGGCACCAAAGTTGTTGCTG
RT-GAPDH-F (5′–3′)	ATCCTGGGCTACACTGAGGA
RT-GAPDH-R (5′–3′)	TGTCGTACCAGGAAATGAGCT

### Western blotting

Frozen pancreas samples were ground in liquid nitrogen, and the resultant powder was solubilized in lysis buffer. The extracts were incubated on ice for 50 min and centrifuged at 12,000 rpm for 10 min at 4 °C. Protein concentrations were calculated using a BCA Protein Assay Kit (Beyotime, Haimen, China). Equal amounts (40 μg) of proteins were separated by sodium dodecyl sulfate polyacrylamide gel electrophoresis on a 4% separating gel, and the protein bands were electrophoretically transferred to a nitrocellulose membrane and blocked in 5% skim milk powder for 2 h at room temperature. The membrane was subsequently incubated with a primary antibody (Amylin-sc-377530-Santa Cruz Biotech., Santa Cruz, CA, 1:100) overnight at 4 °C. The membrane was washed 3 times for 10 min with TBST buffer. Then, the membrane was incubated for 1 h with the secondary antibody diluted 1:2000 with TBST buffer. Finally, the membrane was visualized with the ECL-Plus Western Blotting Kit (Beyotime, Haimen, China).

### H&E staining

Fresh pancreatic tissue was fixed in 4% PFA, embedded in paraffin, and sectioned at 5 μm. The procedure was followed according to the standard method of H&E staining.

### Detection of fasting blood glucose and insulin levels

Blood was collected from the anterior vena cava of the experimental group and the control group every 2 weeks. The animals were strictly fasted for more than 16 h before blood collection. After the blood samples were collected, they were taken back to the laboratory and centrifuged at 3 °C for 3000 rpm for 10 min. The serum was collected, and the levels of fasting blood glucose and fasting insulin in the serum were measured.

### Intravenous glucose tolerance test

After the experimental animals were fasted for 16 h, 50% glucose was injected intravenously at 1.2 mL/kg, and the injection was completed within 3 min. Blood glucose levels were measured at 0, 30, 60, 90, and 120 min.

### Calculation of insulin resistance index (HOMA-IR)

HOMA-IR is an internationally used indicator for assessing an individual's insulin resistance level. The calculation method is as follows:

HOMA-IR = fasting blood glucose (FBG, mmol/L) × fasting insulin (FINS, mU/L)/22.5

### Statistical analysis

All data were expressed as the means ± SEMs, and Student’s *t* test was used for statistical analysis.

## Supplementary information

Supplement table

Supplementary Figure S1

Supplementary Figure Legends

Author-contribution-form

## References

[1] Miley, D. D. & Terezhalmy, G. T. The patient with diabetes mellitus: etiology, epidemiology, principles of medical management, oral disease burden, and principles of dental management. *Quintessence Int.* **36**, 779–795 (2005). 16261794

[2] Collaboration ERF (2011). Diabetes mellitus, fasting glucose, and risk of cause-specific death. New Engl. J. Med..

[3] Chen L, Magliano DJ, Zimmet PZ (2012). The worldwide epidemiology of type 2 diabetes mellitus—present and future perspectives. Nat. Rev. Endocrinol..

[4] Roglic G, Unwin N (2010). Mortality attributable to diabetes: estimates for the year 2010. Diabetes Res. Clin. Pract..

[5] Association AD (2013). Diagnosis and classification of diabetes mellitus. Diabetes Care.

[6] Kokil GR, Veedu RN, Ramm GA, Prins JB, Parekh HS (2015). Type 2 diabetes mellitus: limitations of conventional therapies and intervention with nucleic acid-based therapeutics. Chem. Rev..

[7] Drivsholm T, de Fine Olivarius N, Nielsen ABS, Siersma V (2005). Symptoms, signs and complications in newly diagnosed type 2 diabetic patients, and their relationship to glycaemia, blood pressure and weight. Diabetologia.

[8] Meijnikman A (2016). Screening for type 2 diabetes mellitus in overweight and obese subjects made easy by the FINDRISC score. J. Diabetes Complic..

[9] Hamilton MT, Hamilton DG, Zderic TW (2007). Role of low energy expenditure and sitting in obesity, metabolic syndrome, type 2 diabetes, and cardiovascular disease. Diabetes.

[10] Association AD (2000). Type 2 diabetes in children and adolescents. Pediatrics.

[11] Ginter, E. & Simko, V. *Diabetes* 42–50 (Springer, 2013).

[12] Kahn SE (1990). Evidence of cosecretion of islet amyloid polypeptide and insulin by β-cells. Diabetes.

[13] Marzban L, Trigo-Gonzalez G, Verchere CB (2005). Processing of pro-islet amyloid polypeptide in the constitutive and regulated secretory pathways of β cells. Mol. Endocrinol..

[14] Gasa R, Gomis R, Casamitjana R, Novials A (1997). Signals related to glucose metabolism regulate islet amyloid polypeptide (IAPP) gene expression in human pancreatic islets. Regulatory Pept..

[15] Wagner JD (2001). Naturally occurring and experimental diabetes in cynomolgus monkeys: a comparison of carbohydrate and lipid metabolism and islet pathology. Toxicol. Pathol..

[16] Meier JJ (2006). Inhibition of human IAPP fibril formation does not prevent β-cell death: evidence for distinct actions of oligomers and fibrils of human IAPP. Am. J. Physiol. Endocrinol. Metab..

[17] Pithadia, A., Brender, J. R., Fierke, C. A. & Ramamoorthy, A. Inhibition of IAPP aggregation and toxicity by natural products and derivatives. *J. Diabetes Res.* **2016**, 12 (2016). 10.1155/2016/2046327 PMC4662995 26649317

[18] Grimm, J., Heitz, F., Wirth, F. & Welt, T. Novel compounds capable of antagonizing islet amyloid polypeptide (iapp) induced beta-cell damage and impaired glucose tolerance (Google Patents, 2018).

[19] Javeed N, Matveyenko AV (2018). Circadian etiology of type 2 diabetes mellitus. Physiology.

[20] Liao F, Yoon H, Kim J (2017). Apolipoprotein E metabolism and functions in brain and its role in Alzheimer's disease. Curr. Opin. Lipidol..

[21] Chiti F, Dobson CM (2017). Protein misfolding, amyloid formation, and human disease: a summary of progress over the last decade. Annu. Rev. Biochem..

[22] Camargo DCR (2017). The redox environment triggers conformational changes and aggregation of hIAPP in Type II Diabetes. Sci. Rep..

[23] Mukherjee A, Morales-Scheihing D, Butler PC, Soto C (2015). Type 2 diabetes as a protein misfolding disease. Trends Mol. Med..

[24] Meurens F, Summerfield A, Nauwynck H, Saif L, Gerdts V (2012). The pig: a model for human infectious diseases. Trends Microbiol..

[25] Roura E (2016). Critical review evaluating the pig as a model for human nutritional physiology. Nutr. Res. Rev..

[26] Weinberg MA, Bral M (1999). Laboratory animal models in periodontology. J. Clin. Periodontol..

[27] Lunney JK (2007). Advances in swine biomedical model genomics. Int. J. Biol. Sci..

[28] Pond, W. G., Church, D. C., Pond, K. R. & Schoknecht, P. A. *Basic Animal Nutrition and Feeding*. (John Wiley & Sons, 2004).

[29] Guilloteau P, Zabielski R, Hammon HM, Metges CC (2010). Nutritional programming of gastrointestinal tract development. Is the pig a good model for man?. Nutr. Res. Rev..

[30] Rees D, Alcolado J (2005). Animal models of diabetes mellitus. Diabet. Med..

[31] King AJ (2012). The use of animal models in diabetes research. Br. J. Pharmacol..

[32] Caillon, L., Hoffmann, A. R., Botz, A. & Khemtemourian, L. Molecular structure, membrane interactions, and toxicity of the islet amyloid polypeptide in type 2 diabetes mellitus. *J. Diabetes Res.* **2016**, 13 (2016). 10.1155/2016/5639875 PMC4655289 26636105

[33] N. Fawver J (2014). Islet amyloid polypeptide (IAPP): a second amyloid in Alzheimer's disease. Curr. Alzheimer Res..

[34] Fu Z, R Gilbert E, Liu D (2013). Regulation of insulin synthesis and secretion and pancreatic Beta-cell dysfunction in diabetes. Curr. Diabetes Rev..

[35] Brender JR, Salamekh S, Ramamoorthy A (2011). Membrane disruption and early events in the aggregation of the diabetes related peptide IAPP from a molecular perspective. Acc. Chem. Res..

[36] Jayasinghe SA, Langen R (2007). Membrane interaction of islet amyloid polypeptide. Biochim. Biophys. Acta.

[37] Brender JR (2008). Amyloid fiber formation and membrane disruption are separate processes localized in two distinct regions of IAPP, the type-2-diabetes-related peptide. J. Am. Chem. Soc..

[38] Zraika S (2009). Oxidative stress is induced by islet amyloid formation and time-dependently mediates amyloid-induced beta cell apoptosis. Diabetologia.

[39] De Koning E, Bodkin N, Hansen B, Clark A (1993). Diabetes mellitus in Macaca mulatta monkeys is characterised by islet amyloidosis and reduction in beta-cell population. Diabetologia.

[40] Betsholtz C (1990). Structure of cat islet amyloid polypeptide and identification of amino acid residues of potential significance for islet amyloid formation. Diabetes.

[41] Zhao J, Yu X, Zhao C, Wang Q, Zheng J (2011). Molecular dynamics simulations of human islet amyloid polypeptide (IAPP) oligomers and their interactions with lipid bilayers. Biophys. J..

[42] Matveyenko AV, Butler PC (2006). Islet amyloid polypeptide (IAPP) transgenic rodents as models for type 2 diabetes. ILAR J..

[43] Nelson RW, Reusch CE (2014). Classification and etiology of diabetes in dogs and cats. J. Endocrinol..

[44] Paulsson JF, Benoit-Biancamano M-O, Schäffer L, Dahl K (2011). Ferret islet amyloid polypeptide (IAPP): characterization of in vitro and in vivo amyloidogenicity. Amyloid.

[45] Christmanson L (1993). Islet amyloid polypeptide in the rabbit and European hare: studies on its relationship to amyloidogenesis. Diabetologia.

[46] Westermark GT, Gebre-Medhin S, Steiner DF, Westermark P (2000). Islet amyloid development in a mouse strain lacking endogenous islet amyloid polypeptide (IAPP) but expressing human IAPP. Mol. Med..

[47] Li SJ (2015). Development of a dietary‐induced metabolic syndrome model using miniature pigs involvement of AMPK and SIRT 1. Eur. J. Clin. Investig..

[48] Kuzmuk KN, Schook LB (2011). Pigs as a model for biomedical sciences. Genet. Pig.

[49] Thim T (2010). Familial hypercholesterolaemic downsized pig with human-like coronary atherosclerosis: a model for preclinical studies. EuroIntervention.

[50] Dufrane D, Goebbels R, Fdilat I, Guiot Y, Gianello P (2005). Impact of porcine islet size on cellular structure and engraftment after transplantation: adult versus young pigs. Pancreas.

[51] Guz Y (1995). Expression of murine STF-1, a putative insulin gene transcription factor, in beta cells of pancreas, duodenal epithelium and pancreatic exocrine and endocrine progenitors during ontogeny. Development.

[52] Kim A (2009). Islet architecture: a comparative study. Islets.

[53] Kim W, Egan JM (2008). The role of incretins in glucose homeostasis and diabetes treatment. Pharmacol. Rev..

[54] Xie Z (2017). Optimization of a CRISPR/Cas9-mediated knock-in strategy at the porcine Rosa26 locus in porcine foetal fibroblasts. Sci. Rep..

[55] Wang K (2017). CRISPR/Cas9-mediated knockout of myostatin in Chinese indigenous Erhualian pigs. Transgenic Res..

