# Supplementary material for: Preparation of a new type 2 diabetic miniature pig model via the CRISPR/Cas9 system
Source: Cell Death Dis. 2019 Oct 28;10(11):823. doi: 10.1038/s41419-019-2056-5 (PMC6817862; doi:10.1038/s41419-019-2056-5)
Supplement: Supplementary file 1 — Supplement table [file 41419_2019_2056_MOESM1_ESM.docx]

**Supplementary Table 1**

Primers for PCR amplification of OTS.

| Primers | Sequences (5' to 3') | Amplicon (bp) |
| --- | --- | --- |
| pIAPP- sgRNA#1-OTS1 | GGAGAGCTTCTGGCCCAAAT | 531 |
|  | TGAAAACCCACCAGGCTTGT |  |
| pIAPP- sgRNA#1-OTS2 | CTCTGGGTTGAGCCTGAAAG | 572 |
|  | CTGTGCTCCAGCAAATGAGA |  |
| pIAPP - sgRNA#1-OTS3 | GAAAAGCGAAGACGGAGAGA | 547 |
|  | TCCTGATGGACAGCTCCTCT |  |
| pIAPP - sgRNA#1-OTS4 | ATAGCAGTGCGGGATGATTC | 507 |
|  | AGGAACACCCACAGCTTCAG |  |
| pIAPP - sgRNA#1-OTS5 | GGTAAGTCTCCCCCTGGTTC | 546 |
|  | TGGGCTGTATTGAAGTGCTG |  |
| pIAPP - sgRNA#1-OTS6 | AGGTGAATGGAGGCAACTGA | 539 |
|  | CCTTTTCCCCAGTACAGCAA |  |
| pIAPP - sgRNA#2-OTS1 | AAATGTGGCGTCAAAGGAAG | 535 |
|  | AGGTGGTAGGCAGGGAGTTT |  |
| pIAPP - sgRNA#2-OTS2 | TTTGCCAATCAAGTCCCACT | 596 |
|  | CCAAGTTGTTAATAGCCCGTTT |  |
| pIAPP - sgRNA#2-OTS3 | AGGAGGGAAAAGGCAGATGT | 570 |
|  | CCTCTCCCTGTGGAGTCAAG |  |
| pIAPP - sgRNA#2-OTS4 | ACACAAAAAGAGCCCTGGTC | 592 |
|  | CCTTTCAGGAAAACCCAACA |  |
| pIAPP –sgRNA#2-OTS5 | TGTTCTGTTGTGGGTGCATT | 596 |
|  | CGGGACAGCCTATATCTCCA |  |
| pIAPP –sgRNA#2-OTS6 | GTGCTGCGTGAAGGAAGAAT | 567 |
|  | TTTTTCTTCATGGGGCTTTG |  |
| pIAPP –sgRNA#2-OTS7 | TGATGAATGGCAACTCTGGA | 524 |
|  | TTGTCCCGTTTAAAGCCAAT |  |
| pIAPP –sgRNA#2-OTS8 | CAGTGCTGTTGGTCCAGAGA | 506 |
|  | CAAAAAGGCGAAGAGAGGAA |  |
| pIAPP –sgRNA#2-OTS9 | TCAGAAGTTCAACCCCAGGC | 564 |
|  | CACATGCTATGCTGGGCCTA |  |
