# Supplementary figures and images for: Preparation of a new type 2 diabetic miniature pig model via the CRISPR/Cas9 system
Source: Cell Death Dis. 2019 Oct 28;10(11):823. doi: 10.1038/s41419-019-2056-5 (PMC6817862; doi:10.1038/s41419-019-2056-5)

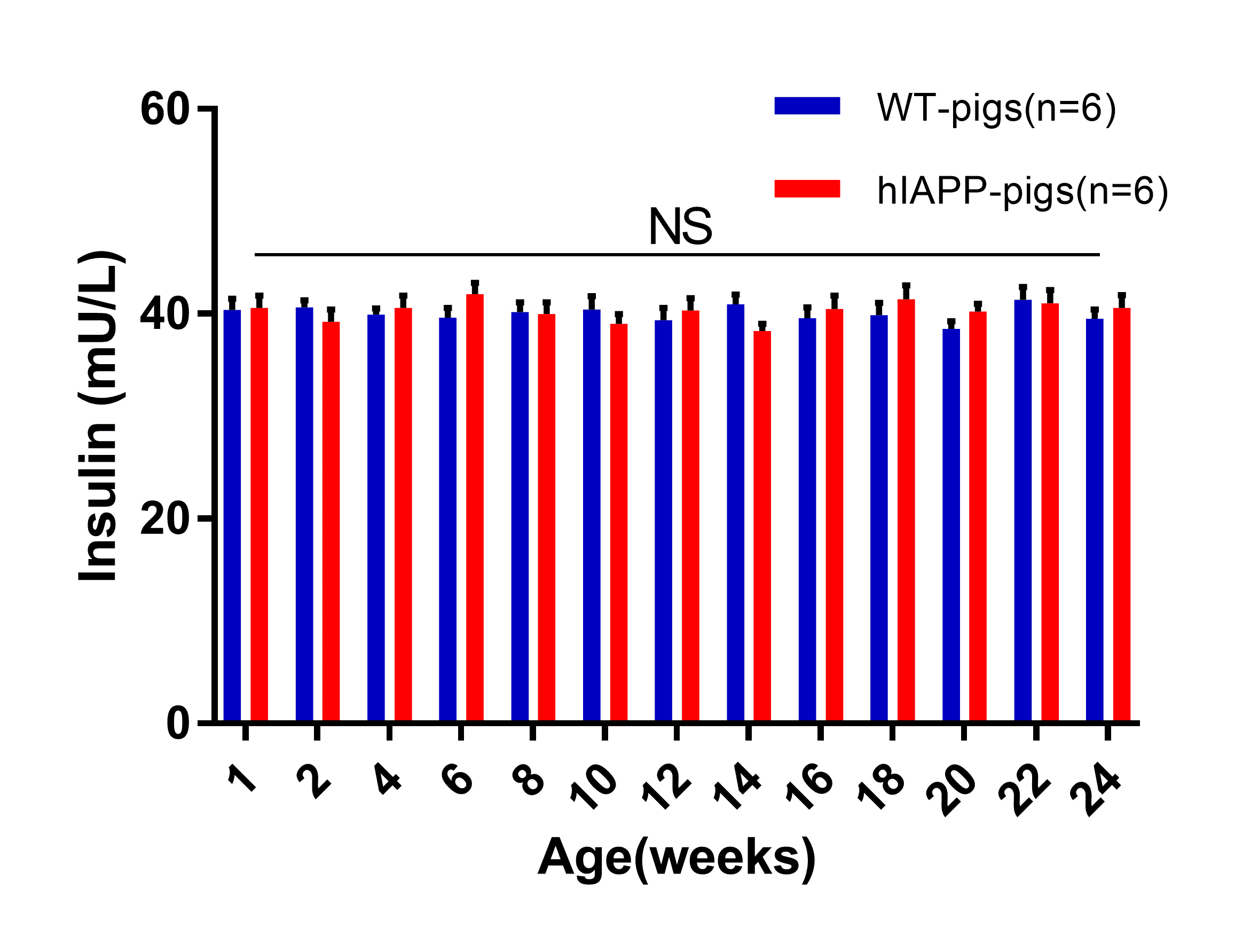

Supplement: Supplementary file 2 — Supplementary Figure S1 [file 41419_2019_2056_MOESM2_ESM.png]
