# Supplementary material for: Preparation of a new type 2 diabetic miniature pig model via the CRISPR/Cas9 system
Source: Cell Death Dis. 2019 Oct 28;10(11):823. doi: 10.1038/s41419-019-2056-5 (PMC6817862; doi:10.1038/s41419-019-2056-5)
Supplement: Supplementary file 3 — Supplementary Figure Legends [file 41419_2019_2056_MOESM3_ESM.docx]

**Supplementary Table 1**

Primers for PCR amplification of OTS.

**Supplementary Figure S1**

Fasting insulin levels of the hIAPP and WT pigs from 3 days to 24 weeks. P < 0.05 was considered statistically significant, *, P < 0.05, **, P < 0.01, ***, P < 0.005. NS, not significant.
