# Supplementary material for: Preparation of a new type 2 diabetic miniature pig model via the CRISPR/Cas9 system
Source: Cell Death Dis. 2019 Oct 28;10(11):823. doi: 10.1038/s41419-019-2056-5 (PMC6817862; doi:10.1038/s41419-019-2056-5)
Supplement: Supplementary file 4 — Author-contribution-form [file 41419_2019_2056_MOESM4_ESM.pdf]

DECLARATION OF CONTRIBUTIONS TO ARTICLE

ADMC

Manuscript Number: CDDIS-19-1913R

Journal Name: *Cell Death & Disease* (the 'Journal')

Proposed Title of the Contribution: Preparation of a new type 2 diabetic miniature pig model via the CRISPR / Cas9 system (the 'Contribution)

Author(s): Xiaodong Zou, Hongsheng Ouyang, Tingting Yu, Xue Chen, Daxin Pang, Xiaochun Tang, Chengzhen Chen (the 'Authors')

For all *CDDis* articles, each person named as an author in the published version must be able to show he or she has contributed substantially to the article.

Authorship credit should be based on 1) substantial contributions to conception and design, acquisition of data, or analysis and interpretation of data; 2) drafting the article or revising it critically for important intellectual content; and 3) final approval of the version to be published. Authors should meet conditions 1, 2 and 3.

Any person who cannot be shown to have made a substantial contribution to the article cannot be listed as an author in the final version. The name of any person who is deemed to have made a minor contribution can, however, appear in the Acknowledgments section of the article.

Please complete the table below to indicate the contributions of all named authors to the manuscript.

Author Full Name:	Specification of Contribution to the Manuscript:
Xiaodong Zou	Conceptualization, Methodology, Writing – original draft
Hongsheng Ouyang	Conceptualization, Methodology, Writing – original draft
Tingting Yu	Investigation, Methodology
Xue Chen	Investigation, Methodology
Daxin Pang	Writing – review & editing
Xiaochun Tang	Writing – review & editing
Chengzhen Chen	Writing – original draft, Writing – review & editing

Please complete the table below to indicate the contributions of all named authors to the figures.

Figure 1:

Design and drawing: Xiaodong Zou, Tingting Yu and Xue Chen.
Layout and modification: Hongsheng Ouyang and Chengzhen Chen.

Figure 2:

Design and drawing: Xiaodong Zou, Tingting Yu and Xue Chen.
Layout and modification: Hongsheng Ouyang and Chengzhen Chen.

Figure 3:

Design and drawing: Xiaodong Zou, Tingting Yu and Xue Chen.
Layout and modification: Hongsheng Ouyang and Chengzhen Chen.

Figure 4:

Design and drawing: Xiaodong Zou, Tingting Yu and Xue Chen.
Layout and modification: Hongsheng Ouyang and Chengzhen Chen.

Figure 5:

Design and drawing: Xiaodong Zou, Tingting Yu and Xue Chen.
Layout and modification: Hongsheng Ouyang and Chengzhen Chen.

Figure 6:

Signed for and on behalf of the Author(s):	Print Name:	Date:
Chengzhen Chen	Chengzhen Chen	September 25, 2019
